# The effects of wearing face masks on the perception and mood of male healthy male adults during treadmill running: A pilot study

**DOI:** 10.14814/phy2.16036

**Published:** 2024-05-16

**Authors:** Kento Hidaka, Shogo Sonoda, Taiki Yamaguchi, Yuka Kose, Kazuki Hyodo, Kazuto Oda, Hiroaki Eshima

**Affiliations:** ^1^ Department of International Tourism, Faculty of Human and Social Studies Nagasaki International University Sasebo Nagasaki Japan; ^2^ National Institute of Technology Sasebo College Sasebo Nagasaki Japan; ^3^ Physical Fitness Research Institute Meiji Yasuda Life Foundation of Health and Welfare Tokyo Japan; ^4^ Department of Health and Nutrition, Faculty of Health Management Nagasaki International University Sasebo Nagasaki Japan

**Keywords:** long distance runners, running, surgical face masks

## Abstract

In the past few years, the face mask has been recommended for the prevention of exposing others to COVID‐19. Wearing a face mask may have the potential to increase dyspnea and discomfort during exercise; however, controversy exists on whether wearing face masks during exercise affects exercise performance, perception, and mood in runners. We investigated the physiological and perceptual responses of healthy male adults who had experienced long‐distance running while exercising at different intensities. Nine healthy young adults who were long‐distance runners wearing surgical face mask conducted an incremental treadmill protocol. The protocol was three 6‐min stages (20%, 40%, and 60% of maximal heart rate, respectively). The rating of perceived exertion (RPE) and the feeling scale (FS) were measured. RPE was higher in mask condition than in unmask condition (No mask vs. Face mask, light; 8.22 vs. 8.78, *p* = 0.615, middle; 10.00 vs. 10.78, *p* = 0.345, high; 12.33 vs. 13.67, *p* = 0.044.), while FS was not different between conditions. The present study shows that wearing a mask may increase rating of perceived exertion and discomfort when the exercise intensity exceeds a certain threshold in healthy male adults who have experienced long‐distance running.

## 1 INTRODUCTION

Over recent years, the coronavirus disease 2019 (COVID‐19) has rapidly spread around the world and led to the pandemic; thus some governments have strictly mandated to wear masks to minimize the risk of transmission of COVID‐19 (Jefferson et al., 2023). Wearing a face mask during exercise is no exception and thereby it may increase respiratory resistance (Ade et al., 2021). However, many studies demonstrate wearing a face mask did not affect dyspnea, pulse rate, and percutaneous arterial oxygen saturation (SpO_2_) during exercise in young healthy adults (Fukushi et al., 2021; Rudi et al., 2022; Shaw et al., 2020; Zhang et al., 2021). Indeed, a recent systematic review showed that the effect on performance of wearing a face mask during exercise appeared to be small in healthy individuals (Zheng et al., 2023). On the other hand, a previous study demonstrates wearing a face mask reduced maximum performance, oxygen consumption and minute ventilation in well‐trained athletes including road cyclists, mountain bikers, and triathletes (Egger et al., 2022). Another study shows wearing a mask negatively impacts subjective feelings of training in track and field athletes including sprinters and long jumpers (Dantas et al., 2023).

Long‐distance runners have higher cardiopulmonary function than healthy adults and excessive respiratory resistance with face masks may be expected (Prado et al., 2021). Indeed, a previous study demonstrated the effect of wearing face mask with regards to exercise capacity such as running economy in males, as well as an increase in rating of perceived exertion (RPE) and respiratory discomfort (Prado et al., 2021). Another report shows no differences in maximal exercise levels in runners for physiological capacities such as ventilation, tidal volume, and breathing frequency with or without wearing a face mask (Evans & Potteiger, 1995). Thus, there is no current consensus on the effects of wearing a face mask on exercise performance in runners.

A recent study demonstrated RPE was significantly higher when exercising vigorously in healthy adults wearing masks, suggesting that wearing a face mask may influence perception and mood during intensity‐dependent exercise (Poon et al., 2021). Indeed, a previous study demonstrated an increased RPE was reported with mask use on vigorous exertion in runners (Prado et al., 2021). However, there is no current evidence to support the impact of exercise intensity on both the perceptions and the moods of long‐distance runners while wearing a face mask.

Based on the previous research studies, we hypothesized that wearing a surgical mask during exercise of different intensities in long‐distance runners might lead to negative perceptions and moods because athletes tend to have higher cardiopulmonary function and respiratory resistance while wearing face masks. In addition, we examined whether wearing a face masks causes changes in exercise performance such as running speed.

## 2 METHOD

### 2.1 Participants

Nine young male recreational long‐distance runners (Table 1) participated in this study. All participants had kept habitual running for at least 90 km/week for the year, and each of the runners had experiences ranging between 5 and 13 years. All participants were healthy, free from lower‐extremity injuries for the past 12 months, and no subjective symptoms that would impede running at the time of measurement. The participants were asked not to perform any strenuous exercise for at least 24 h before the measurement. They fasted after waking up and took only water before measurement.

**TABLE 1:** Characteristics of the participants.

Variable	Runners
*n*	9
Age (years)	20.0 ± 0.9
Body height (m)	1.71 ± 0.05
Body weight (kg)	54.4 ± 4.7
BMI (kg/m^2^)	18.6 ± 1.0
Running experience (years)	9.6 ± 2.3
Running distance (km/week)	104.4 ± 8.3

*Note*: Values are the means ± SD.

### 2.2 Experimental trials

The participants completed two experimental trials, both with and without a face mask, on a standardized treadmill (O_2_road, Takei Sci. Instruments Co., Niigata, Japan). A surgical face mask (Safe+Mask1Premier, Medicom, Kobe, Japan) with earloops, while dye free non–woven inner layer provides additional protection was used in this study (Fukushi et al., 2021). These surgical face masks are widely used in hospitals, in gyms, and in everyday life in Japan. It was also found that a cloth face mask increased the degree of dyspnea more than wearing a surgical face mask during exercise (Fukushi et al., 2021). Participants followed the same incremental treadmill protocol in each trial, with three 6‐min stages, for a total of 18 min, at 20%, 40%, and 60% estimated maximum heart rate (HR), respectively. HR was monitored throughout the experimental period using a Polar H7 (Polar Electro Oy, Kempele, Finland). Data were recorded at the baseline and across different intensities of exercise (i.e., at the end of each 6‐min stage). For running speed, the average of the last 15 s of running speed were used at the end of each stage. To estimate the exercise intensity, we calculated the target heart rate for each subject according to Karvonen formula (Karvonen et al., 1957).

No external stimuli or verbal encouragement were provided during the trials, and the trials were performed over a 1‐week interval. Before and after exercise, we confirmed that the SpO_2_ levels remained within the normal physiological range (95%–100%) throughout the session, indicating no sign of hypoxia. The room temperature and barometric pressure when we were conducting trials. The room's barometric pressure was in the range of 1023.0–1025.8 hPa and the room's altitude was 4.6 m. The room temperature was set at 26°–28°.

### 2.3 Rating of perceived exertion and feeling scale

RPE was assessed using the Borg scale (Shariat et al., 2018). The scale ranged from 6 to 20, with anchors ranging from “No exertion at all” (score, 6) to “Maximal exertion” (score, 20). To assess the participant's mood during the exercise, we used the feeling scale (FS) comprising of a single item (Hyodo et al., 2021). The item assesses how the participant currently feels (pleasant–unpleasant) about the exercise on an 11‐point bipolar scale with scores ranging from −5 (very bad) to +5 (very good). We calculated the mean FS score during exercise to assess whether the mood was pleasant while exercising. The RPE and the FS were obtained verbally from each participant at the baseline and the end of each stage. The area under the curve was determined by summation at the baseline and the end of each stage.

### 2.4 Statistical analysis

Statistical analyses were performed with Prism v.9.0 (GraphPad Software, San Diego, CA, USA). Data were presented as means and standard deviations. Paired sample *t*‐tests (two‐sided) were used to compare two groups. For multiple comparisons, two‐way ANOVA were performed followed by appropriate posthoc tests corrected for multiple comparisons. For all tests, *p* < 0.05 was considered statistically significant.

## 3 RESULTS

**FIGURE 1:**
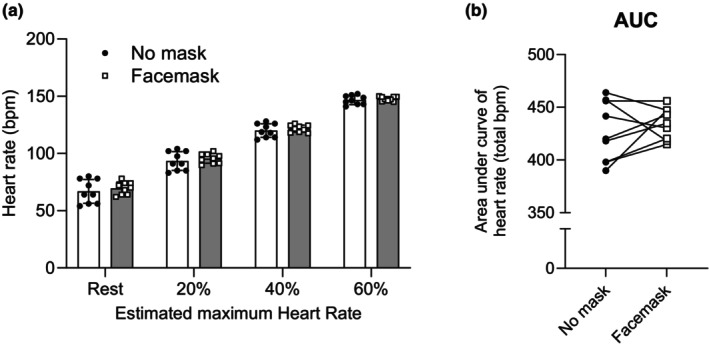
Mean changes (a) and area under the curve (AUC) (b) in heart rate during exercise with and without a surgical mask. Values are means ± SD (*n* = 9).

**FIGURE 2:**
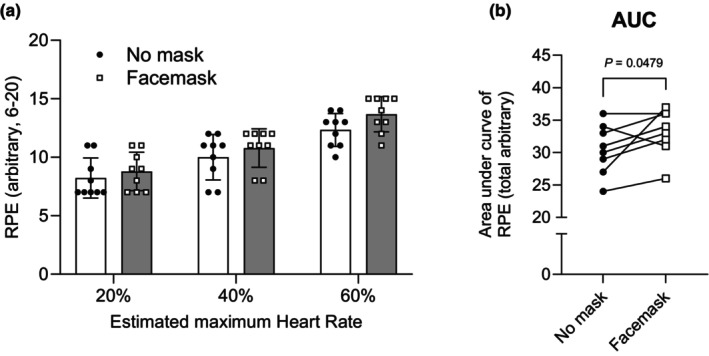
Mean changes (a) and area under the curve (AUC) (b) in rating of perceived exertion (RPE) during exercise with and without a surgical mask. Values are means ± SD (*n* = 9).

**FIGURE 3:**
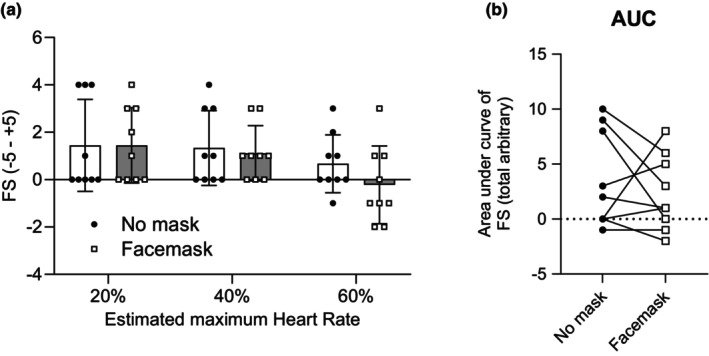
Mean changes (a) and area under the curve (AUC) (b) in feeling scale (FS) during exercise with and without a surgical mask. Values are means ± SD (*n* = 9).

**FIGURE 4:**
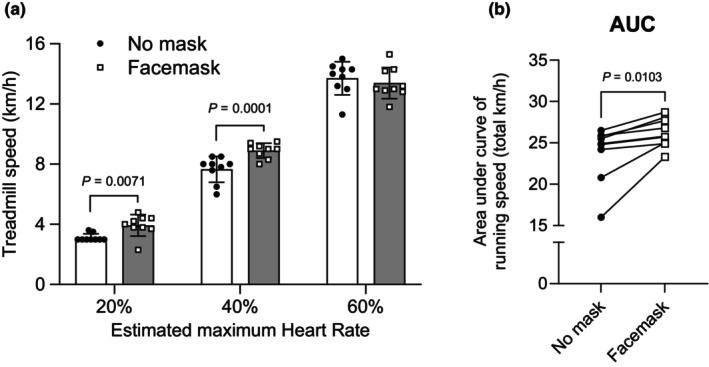
Mean changes (a) and area under the curve (AUC) (b) in running speed during exercise with and without a surgical mask. Values are means ± SD (*n* = 9).

Figure 1a shows HR data at rest and at each exercise stage. Two‐way ANOVA showed a significant main effect of intensity (*F*(3, 24) = 2856, *p* < 0.0001, *ηp* ^2^ = 0.998), there were no significant main effect of condition (*F*(1, 8) = 0.68, *p* = 0.435, *ηp* ^2^ = 0.473) and interaction (*F*(3, 24) = 0.068, *p* = 0.573, *ηp* ^2^ = 0.078). Posthoc analysis of the main effect of intensity showed significant differences between all comparisons (all *p* < 0.0001). There was no significant difference in the area under curve (AUC) of HR between the masked and unmasked conditions (*t*(8) = 0.8224, *p* = 0.4347, *d =* 0.2741) (Figure 1b). These results indicate that HR increased gradually with increasing exercise intensity regardless of conditions, and there was no difference in HR at each stage. Figure 2 shows the RPE data as perceptual measurements during exercise. As with HR, there were significant main effect of intensity (*F*(2, 16) = 30.52, *p* < 0.0001, *ηp* ^2^ = 0.914), and main effect of condition (*F*(1, 8) = 5.447, *p* = 0.048, *ηp* ^2^ = 0.383), but no significant interaction (*F*(2, 16) = 0.671, *p* = 0.525, *ηp* ^2^ = 0.077). Posthoc analysis of the main effect of intensity showed significant differences between all comparisons (all *p* < 0.0001) (Figure 2a). The AUC of RPE was higher in mask condition than in unmask condition (*t*(8) = 2.334, *p* = 0.0479, *d =* 0.788) (Figure 2b). These results indicate that RPE increased gradually with exercise intensity regardless of conditions. Figure 3 represents the FS (Figure 3a) and AUC (Figure 3b). Two‐way ANOVA showed significant main effect of intensity (*F*(2, 16) = 5.793, *p* = 0.0128, *ηp* ^2^ = 0.134), but no significant main effect of condition (*F*(1, 8) = 0.49, *p* = 0.501, *ηp* ^2^ = 0.130) and no significant interaction (*F*(2, 16) = 1.24, *p* = 0.32, *ηp* ^2^ = 0.134). Posthoc analysis of the main effect of intensity showed that FS at 40% HRmax running was significantly higher than at 60% HRmax (*p =* 0.0162). AUC of FS was not different between conditions (*t*(8) = 0.7049, *p* = 0.5009, *d =* −0.234) (Figure 3b). Figure 4 represents the running speed (Figure 4a) and AUC (Figure 4b). We found significant main effect of intensity (*F*(2, 16) = 793.1, *p* < 0.0001, *ηp* ^2^ = 0.996), significant main effect of condition (*F*(1, 8) = 20.76, *p* = 0.0019, *ηp* ^2^ = 0.553), and significant interaction (*F*(2, 16) = 12.92, *p* = 0.0005, *ηp* ^2^ = 0.618). The simple main effect of condition on each intensity showed that running speeds in mask condition were higher at 20% HRmax running (*p* = 0.0071) and 40% HRmax running (*p* = 0.0001) than in unmasked condition. The AUC of running speed was significantly higher in mask condition than in unmask condition (*t*(8) = 3.338, *p* = 0.0103, *d =* 1.113) (Figure 4b).

## 4 DISCUSSION

In this study, we aimed to clarify the effects of wearing a face mask on perception, and mood in long‐distance runners during treadmill running. Previous studies demonstrate that wearing a surgical mask reduces pulmonary function and ventilation at maximal exercise in healthy men (Driver et al., 2022; Fikenzer et al., 2020), suggesting that wearing a surgical mask may negatively affect physiological conditions at a fixed submaximal exercise intensity (Chandrasekaran & Fernandes, 2020). In contrast, a recent study shows that surgical masks had no effect on respiratory functions in three 8‐min cycling trials in healthy adults (Doherty et al., 2021). Indeed, another study shows that wearing a mask does not affect physiological function in the 6‐min three stage of exercise in healthy adults (Poon et al., 2021). These findings suggest that short, light‐intensity exercise does not significantly affect physiological function with wearing a mask in healthy subjects. A previous study shows no differences in RPE during light‐moderate intensity exercise between the mask and unmasked conditions in healthy subjects (Poon et al., 2021). For mood during exercise, light‐intensity exercise could induce a more pleasant mood during exercise than moderate‐intensity exercise (Ekkekakis et al., 2011). The present study has newly shown that wearing mask affects perceptual and mood during exercise at light‐moderate intensity in healthy male adults who had experienced long‐distance runners.

Previous studies show that greater dyspnea with surgical face masks than without masks in healthy subjects during exercise (Fikenzer et al., 2020; Hopkins et al., 2021). In contrast, another study shows that wearing a surgical mask at a low to moderate did not increase dyspnea in healthy subjects during 1 h treadmill exercise (Roberge et al., 2012). Indeed, wearing a mask did not affect worsened dyspnea at any exercise intensity in healthy subjects (Shaw et al., 2020). The present study did not measure the effect of wearing mask on perceptual and mood during exercise for more than 18 min, but we expect wearing mask would affect perceptual and mood in long distance runners during moderate‐intensity exercise for at least 1 h.

We also examined the effect of wearing a face mask in running speed. A previous study showed that wearing a mask decreased running speed and exercise volume during maximal‐intensity aerobic endurance testing (Slimani et al., 2022). We expected that wearing a mask would cause dyspnea and as a result, less exercise volume, but the data shows the running speed increased (Figure 4a,b). It may suggest runners are accustomed to running exercises on a daily basis, so it is speculated that wearing a mask may be necessary for more exercise volume during low‐intensity exercise; therefore further studies are needed in order to examine the impact of wearing a mask on running exercise performance in runners. In addition, the increased RPE during exercise may be the result of the increased treadmill speed, further study should be examined by another exercise protocol such as the exhaustion test.

This study has some limitations. First, there were no data comparisons for runners. Second, there was no data for respiratory function such as oxygen uptake. In recent years, subjects in this study have been wearing masks on a daily basis due to COVID‐19, so they might be constantly exposed to a hypoxic environment. In addition, runners daily run more distances of 18 min, so time may be an important factor as well as the intensity in this study. Third, because of the small sample size in this study, statistical power might be too low to detect the difference in the effects between no mask and masked conditions. Fourth, the present study examined only male subjects. There were no differences in the assessment of hemodynamics wearing a face mask between men and women during exercise (Ahmadian et al., 2022). However, our data may not be generalized to women. Further studies with a larger sample size, controlling for inactive people and women are needed to further clarify the effect of masks during exercise.

In summary, we found that wearing a face mask affect perceptions and mood during exercise at light‐moderate intensity in long‐distance runners. This finding is useful for understanding the effects of exercising in confined spaces such as gyms where individuals are susceptible to COVID‐19 (Jang et al., 2020). Furthermore, wearing a surgical face mask increases the running speed at low‐intensity exercise in healthy male adults who had experienced long‐distance runners, suggesting that wearing a mask may be utilized to improve running performance.

## AUTHOR CONTRIBUTIONS

Hiroaki Eshima conceived and designed the research; Kento Hidaka performed the experiments; Kento Hidaka, Shogo Sonoda, Taiki Yamaguchi, Yuka Kose, Kazuki Hyodo and Hiroaki Eshima the analyzed data; Kento Hidaka, Shogo Sonoda, Taiki Yamaguchi, Kazuki Hyodo, Kazuto Oda and Hiroaki Eshima interpreted results of the experiments; Shogo Sonoda, Taiki Yamaguchi and Hiroaki Eshima prepared figures; Hiroaki Eshima, Yuka Kose and Kazuki Hyodo drafted the manuscript; Kento Hidaka, Shogo Sonoda, Taiki Yamaguchi, Yuka Kose, Kazuki Hyodo, Kazuto Oda and Hiroaki Eshima, approved the final version of the manuscript.

## FUNDING INFORMATION

This study was supported by Meiji Yasuda Life Foundation of Health and Welfares.

## CONFLICT OF INTEREST STATEMENT

The author declares that the research was conducted in the absence of any commercial or financial relationships that could be construed as a potential conflict of interest.

## ETHICS STATEMENT

The study was conducted in accordance with the Declaration of Helsinki, and its protocol was approved by the Ethics Committee of the Nagasaki International University (approval number: F‐20). All participants gave their written informed consent and experimental trials were conducted at the Exercise Physiology Laboratory, Department of Health and Nutrition, Faculty of Health Management, Nagasaki International University.
