# Correction to The effects of wearing face masks on the perception and mood of male healthy male adults during treadmill running: A pilot study

**DOI:** 10.14814/phy2.16111

**Published:** 2024-06-12

Hidaka K, Sonoda S, Yamaguchi T, Kose Y, Hyodo K, Oda K, Eshima H. The effects of wearing face masks on the perception and mood of male healthy male adults during treadmill running: A pilot study. *Physiol Rep*. 2024 12(10):e16036.

The title of “The effects of wearing face masks on the perception and mood of male healthy male adults during treadmill running: A pilot study” was incorrect. This should have read: “The effects of wearing face masks on the perception and mood of healthy male adults during treadmill running: A pilot study.”

We apologize for this error.

